# Gut microbiome and clinical and lifestyle host factors associated with recurrent positive RT-PCR for SARS-CoV-2

**DOI:** 10.3389/fcimb.2024.1494193

**Published:** 2024-12-18

**Authors:** Cristina Jiménez-Arroyo, Natalia Molinero, Carlos Sabater, Abelardo Margolles, Laura Carmen Terrón-Camero, Eduardo Andrés-León, Manuel Ramos, Margarita del Val, M. Victoria Moreno-Arribas

**Affiliations:** ^1^ Department of Food Biotechnology and Microbiology, Institute of Food Science Research (CIAL), CSIC-UAM, Madrid, Spain; ^2^ Department of Microbiology and Biochemistry of Dairy Products, Dairy Research Institute of Asturias (IPLA-CSIC), Villaviciosa, Asturias, Spain; ^3^ Functionality and Ecology of Beneficial Microbes (MicroHealth) Group, Health Research Institute of Asturias (ISPA), Oviedo, Asturias, Spain; ^4^ Bioinformatics Unit, Institute of Parasitology and Biomedicine “López-Neyra” CSIC (IPBLN-CSIC), Granada, Spain; ^5^ Viral Immunology Unit, Severo Ochoa Molecular Biology Center (CSIC-UAM), Madrid, Spain

**Keywords:** persistent SARS-CoV-2 PCR positivity, shotgun metagenomics, microbiome, host factors, multivariate analysis

## Abstract

**Background:**

SARS-CoV-2 and COVID-19 are still active in the population. Some patients remained PCR-positive for more than 4 weeks, called “persistently PCR-positive”. Recent evidence suggests a link between the gut microbiota and susceptibility to COVID-19, although no studies have explored persistent PCR conditions. We aimed to evaluate the relationship between persistent positive SARS-CoV-2 RT-PCR, the gut microbiome, and individual host determinants.

**Methods:**

A shotgun metagenomic analysis was conducted on fecal samples from 28 individuals affected by COVID-19. Patients were divided into two groups: those who had cleared the virus within 30 days (designated as the control group) (n = 15), and those who remained PCR-positive beyond 30 days (called the PCR+ group) (n = 13). We also investigated the correlation between prolonged viral clearance and several additional factors, including clinical parameters, immune responses, microbial metabolites, and dietary habits.

**Results:**

The composition and functionality of the microbiome varied based on the duration of positivity as determined by PCR. Compared to the control group, the persistent PCR+ group exhibited elevated pathogen levels and augmented diversity in functional gene families (p-value < 0.05). A multi-omics analysis integrating metagenomics, metabolites, and metadata also revealed the specific contribution of certain blood markers in this group, including basophils, IgM, IgG (both general and specific for SARS-CoV-2), and markers of liver damage. Unhealthy diet was identified as a significant factor influencing the duration of PCR positivity.

**Conclusions:**

These findings indicate that the gut microbiome may play a role in delayed viral clearance and persistent positive RT-PCR results. Our study also contributes to the understanding of the role of host factors as mediators linking the gut microbiota and disease outcomes. Further large-scale studies must confirm these data; however, they suggest the relevance of monitoring microbiome changes in the early post-viral years to control SARS-CoV-2 and providing individual healthcare support.

## 1 Introduction

Coronavirus disease 2019 (COVID-19), caused by the severe acute respiratory syndrome coronavirus 2 (SARS-CoV-2) infection, is still active among the population. More than 775 million people across the world had been infected up until August 2024, according to the World Health Organization (WHO) (https://covid19.who.int/). However, there are still many gaps in our knowledge about the virus and the disease, as well as its relation to extrinsic and intrinsic host factors.

The clinical symptoms of COVID-19 can change throughout the course of the illness and, in more severe cases, may lead to acute respiratory distress syndrome. Furthermore, in some individuals, symptoms can persist for months or even years after the acute phase, a condition known as long COVID or PACS (post-acute COVID syndrome) (Scholkmann and May, 2023). One of the proposed mechanisms for long COVID is viral persistence in the body, manifesting either as replicating virus or as fragments of viral RNA and proteins (Al-Aly et al., 2024). It is known that the severe acute respiratory syndrome caused by SARS-CoV-2 has a mean incubation period of 6.57 days, defined as the time from infection to the onset of signs and symptoms, although the virus variant could influence the duration (Wu et al., 2022). An average of 18.29 days from the onset are required for viral RNA clearance from the upper respiratory tract (Okita et al., 2022), whereas viral load peaks at disease onset and shortly after the appearance of symptoms (Motoc et al., 2022). Nevertheless, the duration of viral RNA shedding is variable, and prolonged viral load does not necessarily mean infectiousness (Hong et al., 2020). Most patients with mild and moderate forms of COVID-19 are no longer infectious 10 days after the onset of symptoms (Motoc et al., 2022). However, some patients could test positive for SARS-CoV-2 by reverse transcriptase polymerase chain reaction (RT-PCR) in nasopharyngeal specimens for more than 4 weeks (Aldhaeefi et al., 2021; Long et al., 2021), a condition that is known in the bibliography as “prolonged or persistent positive RT-PCR testing”.

Several studies have shown substantial involvement of the gastrointestinal tract in COVID-­19, as well as the impact of the disease on gut function, including gut microbiome alterations (Chakraborty et al., 2022; Mancabelli et al., 2022; Lin et al., 2023; Rossini et al., 2024). In fact, fecal samples remained positive for SARS-CoV-2 longer than nasopharyngeal samples (Wu et al., 2020; Natarajan et al., 2022; Okita et al., 2022). Moreover, cumulative evidence has shown that gut dysbiosis may be linked to the severity of COVID-19 infection and may persist even after the disease has been resolved (Tian et al., 2021; Bucci et al., 2023; Rossini et al., 2024). COVID-­19 patients had significant alterations in fecal microbiomes compared with noninfected controls, characterized by the enrichment of opportunistic pathogens and the depletion of beneficial commensals, which may vary depending on the viral variant (Liu et al., 2022; Yokoyama et al., 2024). Furthermore, gut microbial imbalances have been found to affect acute inflammation well beyond the gut, including the lung, and has been associated with infection by various respiratory viruses, although the mechanisms underlying microbiome–gut–lung associations are still being explored (Sarkar et al., 2021).

Up until now, very few studies have investigated the factors involved in prolonged positive SARS-CoV-2 RT-PCR testing, especially in mild cases. Hoffman et al. (2021) described a correlation between comorbidities and the number of days required for the virus to clear in nonsevere COVID-19 patients (Hoffman et al., 2021). In addition, Motoc et al. (2022) associated old age, high BMI values, male gender, and cardiac, liver, and renal chronic disease with persistent positive RT-PCR testing in mild and moderate forms of COVID-19 (Motoc et al., 2022). Nevertheless, to the best of our knowledge, no studies have yet explored the relation between the gut microbiome and this condition, nor its relationship with other clinical or lifestyle host factors. Here, we conducted an in-depth analysis of the composition and functional profile of the gut microbiome of persistent SARS-CoV-2-positive RT-PCR subjects using shotgun metagenomics, to investigate the effects of this condition on the human gut bacteriome. As a secondary outcome, we sought to determine whether there was any association between clinical and individual patient characteristics, including dietary patterns, and the difficulty of SARS-CoV-2 clearance.

## 2 Materials and methods

### 2.1 Study cohort, metadata, and sample acquisition

Participants of this prospective cohort study were recruited between January 2021 and January 2022 from the Healthcare Service of the Spanish National Research Council (CSIC). In total, 28 subjects agreed to participate in the study and gave their signed informed consent. Participating in the study was not compensated. The study complied with all relevant international and local regulations, in line with the Declaration of Helsinki, and was approved by the Ethics Committee of the CSIC.

RT‐PCR is the most often used reference standard for detecting SARS-CoV-2 in patients (Struyf et al., 2022). Viral RNA detection from nasopharyngeal samples was performed during the incubation period to confirm the condition, during the infection and the disease course, and after patient discharge to confirm complete clearance of the virus, considering as positive a cycle threshold (Ct) < 34. The recruitment criteria were as follows: (1) individuals diagnosed with SARS-CoV-2 infection by a positive RT-PCR test and who persistently tested positive for > 30 days from the initial positive RT-PCR result (PCR+ group); and (2) age- and sex-matched controls who were diagnosed with SARS-CoV-2 infection as confirmed by an RT-PCR test with a positivity lasting for < 30 days (at least 3 consecutive negative samples) (**Table 1**). The mean duration of positivity for the control group was 25.2 ± 5.3 days, whereas that for the PCR+ group was 59.4 ± 17.1 days. Antibiotics use in the last six months, known complex infections or sepsis, a known history of severe organ failure, bowel surgery in the past 6 months (excluding colonoscopy/procedure related to perianal disease), and the presence of an ileostomy/stoma resulted in exclusion from the study. Additionally, participants with recent use of investigational and/or immunomodulatory agents for treatment of COVID-19 were excluded. Data on COVID-19 symptomatology were recorded. Moreover, subjects answered validated questionnaires relating to their clinical record, lifestyle and dietary patterns, and frequency of consumption of different food groups (Food Frequency Questionnaire, FFQ) (Fernández-Ballart et al., 2010). The FFQ provided data on a total of 118 items, which were subsequently categorized into 15 different food groups. FFQ data were transformed into daily consumption values (g/day) for each item and food group (Fernández-Ballart et al., 2010).

**Table 1:** Characteristics of the cohort involved in the study.

	Total	Control group	PCR+ group	p-value
**Sample size (n)**	28	13	15	
**Gender**				0.706
Women	18 (64.3%)	9 (69.2%)	9 (60.0%)	
Men	10 (35.7%)	4 (30.8%)	6 (40.0%)	
**Age (years)**	49.39 ± 11.28	49.38 ± 12.87	49.40 ± 10.15	0.997
**Body mass index, BMI (kg/m²)**	24.33 ± 3.00	24.15 ± 1.91	24.49 ± 3.77	0.770
**Days with positive RT-PCR testing**	–	25.15 ± 5.26	59.38 ± 17.08	1.19x10^5^
**COVID-19 symptoms and category severity**				0.299
Asymptomatic/Mild	23 (82.1%)	11 (84.6%)	12 (80.0%)	
Moderate	1 (3.6%)	0 (0.0%)	1 (6.6%)	
Severe	4 (14.3%)	2 (15.4%)	2 (13.3%)	
**Presence of preexisting medical conditions**	2 (7.1%)	1 (7.7%)	1 (6.7%)	1
**Presence of chronic treatments**	8 (28.6%)	4 (30.8%)	4 (26.7%)	1
**Current smoking**	3 (10.7%)	1 (7.7%)	2 (13.3%)	1

Continuous variables are expressed as mean ± standard deviation, evaluated through ANOVA or Kruskal-Wallis tests. Categorical variables are displayed as number of volunteers (in %), analyzed using the Fisher’s exact test. Significant differences were only found between groups in terms of the number of the days with positive RT-PCR testing (p-value < 0.05).

Biological samples, specifically blood and fecal matter, were collected from the participants when the RT-PCR test for SARS-CoV-2 yielded a negative result. Stool samples were obtained aseptically by each participant and stored in anaerobic conditions at −80 °C until analysis. Blood samples were collected through standard venepuncture after overnight fasting. Plasma and serum samples were stored at −80 °C.

### 2.2 Blood test and clinical parameters

Serum and plasma biochemical parameters were measured using an automated biochemical auto-analyzer. Humoral (either related or not to COVID-19 disease) and cellular immune status were also analyzed.

### 2.3 Immune response analysis

#### 2.3.1 CD8+ T lymphocyte response

Frozen PBMCs from all donors were thawed in RPMI + 10% human AB serum and cultivated for 24 h. After this time, a fraction was infected as described by Perdiguero et al. (2023) with some exceptions. Briefly, Vaccinia virus (VACV) WR strain as control or VACV recombinants of the SARS-CoV-2 M, N, and S proteins were added at an m.o.i. of 5 and incubated for 1 h at 37 °C to enable virus absorption; then cells were washed and incubated in RPMI + 10% FCS for 5 h of infection. After this time, an equal number of PBMCs from the same donor were added as cell responders and incubated for a further 2 h. Thereafter, Alexa Fluor 488-anti-CD107a antibody (Biolegend, San Diego, CA, USA) and the ER/Golgi transport inhibitors monesin and brefeldin A (Sigma-Aldrich, Saint Louis, MO, USA) were added to enable the accumulation of intracellular cytokines and incubated O/N at 37 °C. The following day, cells were surface stained with APC-anti-CD8α (Biolegend), fixed, and incubated with PE-anti-IFN-γ, BV421-anti-IL-2, and BV605-anti-TNF-α (all from Biolegend) during permeabilization in accordance with conventional procedures. Events were acquired in a BD FACSCanto II flow cytometer and data analyzed using BD FACSDiva software v9.0.

#### 2.3.2 CD4+ T lymphocyte response

Another fraction of the thawed PBMCs from all donors was incubated in RPMI + 10% human AB serum overnight at 37 °C with 2 μl of anti-CD28/CD49 (BD Biosciences, San Jose, CA, USA) co-activator plus recombinant SARS-CoV-2 Mpro, N, or S proteins or with the RBD fragment. Then, cells were incubated with PECy7-anti-CD3, PerCP-anti-CD4, BV605-anti-CD69, APC-anti-CD134, and PE-anti-CD137 antibodies (all from Biolegend) to measure induced activation markers. Events were acquired in a BD FACSCanto II flow cytometer as described by Perdiguero et al. (2023), and data analyzed using BD FACSDiva software v9.0.

### 2.4 Fecal total DNA extraction and shotgun metagenomic sequencing

Fecal samples were subjected to a total DNA extraction optimized protocol, using the QIAamp DNA Stool Mini Kit (Qiagen, Hilden, Germany) following the manufacturer’s instructions with some modifications. Briefly, 0.8 g samples were first diluted in 1/10 PBS and centrifuged for 10 min at maximum speed. Supernatants were filtered (0.22 µm pore) and stored at -80 °C until targeted metabolomic analysis, and pellets were resuspended in 0.2 ml of extraction buffer (20 mM Tris-HCl pH 8.0, 2 mM EDTA, 1.2% Triton 100x and 20 mg/ml lysozyme, 12.5 μg/ml lysostaphin, and 100 U/ml mutanolysin (all from Sigma-Aldrich)). After enzymatic lysis for 1 h at 37 °C, 1 mL of Inhibitex buffer was added, and mechanical disruption was performed in a FastPrep-24^TM^ apparatus (MP Biomedicals, CA, USA). Subsequently, the protocol proceeded according to the manufacturer’s instructions. Before library preparation and sequencing, the quality and quantity of the samples were assessed using the Fragment Analyzer system (Agilent Technologies) in accordance with the manufacturer’s guidelines. Samples with a high-quality DNA profile were further processed. Library preparation was performed using the Illumina DNA Prep Kit (Illumina^®^) from 200 ng of gDNA, and including a negative control. The 100x2 nt paired-end sequencing was conducted on a NovaSeq 6000 sequencer using an S4 flow cell. The total yield was 2.5 Tbp rendering an average of 34.5 M paired-end reads per sample. Demultiplexed FASTQ files were obtained using bcl2fastq software.

### 2.5 Taxonomic profiling and functional categorization

Raw sequences were evaluated using FastQC software; 93.6% of the reads had a quality score > 30, with no adapter accumulation, thus no sample trimming was required. The MetaPhlAn 3.0 tool was applied for taxonomic assignation (Truong et al., 2015). The Decontam tool was used to identify and remove any contaminating microorganisms (Davis et al., 2018). The HUMAnN 3.0 pipeline was implemented for functional assessment (Beghini et al., 2021). A list of abundances of microbial gene families and metabolic pathways was obtained and normalized through HUMAnN3_renorm_table script.

### 2.6 Metabolite quantification from stool samples

Fecal supernatants were used to determine concentrations of different short-chain fatty acids (SCFAs), specifically acetic, butyric, propionic, iso-butyric, valeric, and iso-valeric acids. SCFAs were analyzed by GC–MS as previously described by García-Villalba et al. (2012).

### 2.7 Statistical analysis

Statistical analysis was conducted using RStudio v.2023.06.1 with R v.4.3.1. To assess differences in volunteers’ characteristics and metadata among the study groups, different tests were applied depending on the nature of the data. For categorical variables, the Fisher’s exact test was applied. For numerical variables, normality and homogeneity were first tested using the Shapiro-Wilk and Levene’s tests, respectively. Then, ANOVA was used for parametric data and the Kruskal-Wallis test for nonparametric data. Moreover, the p-values of the metadata variables were adjusted using the Benjamini-Hochberg (BH) method of correction. Statistical significance was considered when p-value or p-adj < 0.05.

Alpha diversity indexes (Chao1, Shannon) and beta diversity estimators (principal coordinates analysis (PCoA) method and Bray-Curtis dissimilarity distances) were calculated using the phyloseq package (McMurdie and Holmes, 2013). Differences between groups in biodiversity metrics were evaluated through parametric, nonparametric, or PERMANOVA test, as appropriate. Results were considered statistically significant when p-value < 0.05. For the differential abundance analysis of taxa, ANCOM and LEfSE, through the microbiomeMarker package (Cao et al., 2022), and DESeq2 (Love et al., 2014) were applied, considering those significant by at least one method. For the differential abundance analysis of pathways, DESeq2 was implemented. For both taxa and pathways, statistical differences were considered when p-adj < 0.05.

To integrate data regarding microbiome composition and functionality, clinical and immunological data, diet (complete data about FFQ, i.e., 118 items), and SCFAs, a multivariate approach was applied using the DIABLO (Data Integration Analysis for Biomarker discovery using Latent variable approaches for Omics studies) method available in the mixOmics package (Rohart et al., 2017), to identify the variables that discriminate the outcome of interest.

## 3 Results

### 3.1 Subject characteristics and metadata

COVID-19 illness is highly variable, ranging from infection with no symptoms through to pneumonia and life-threatening consequences. The groups studied (PCR+ and controls) presented similar characteristics in terms of body mass index (BMI), preexisting medical conditions and chronic treatments, and the severity of COVID-19 symptoms (**Table 1**). With regard to blood markers and lifestyle and individual factors, such as food and nutrients intake, the statistical analysis revealed no significant differences between groups (**Supplementary Tables 1 and 2**). Food groups were selected to assess whether the daily consumption between the PCR+ and control groups was similar. Moreover, the immune response analysis (CD8+ T and CD4+ T lymphocyte response) showed no differences between groups in any marker tested (**Supplementary Table 3**), which is consistent with previous studies on persistent PCR positivity for SARS-CoV-2 in immunodeficient patients (Chan et al., 2023), suggesting no apparent alteration in the immune, humoral or cellular response between persistently PCR-positive and controls. Then, we studied the differences in the gut microbiome between those patients who cleared the virus rapidly versus those who did not.

### 3.2 Differences in gut microbial composition and functionality in persistent positive RT-PCR condition

#### 3.2.1 Differences in gut microbial diversity and composition in persistent positive RT-PCR condition

**Figure 1:**
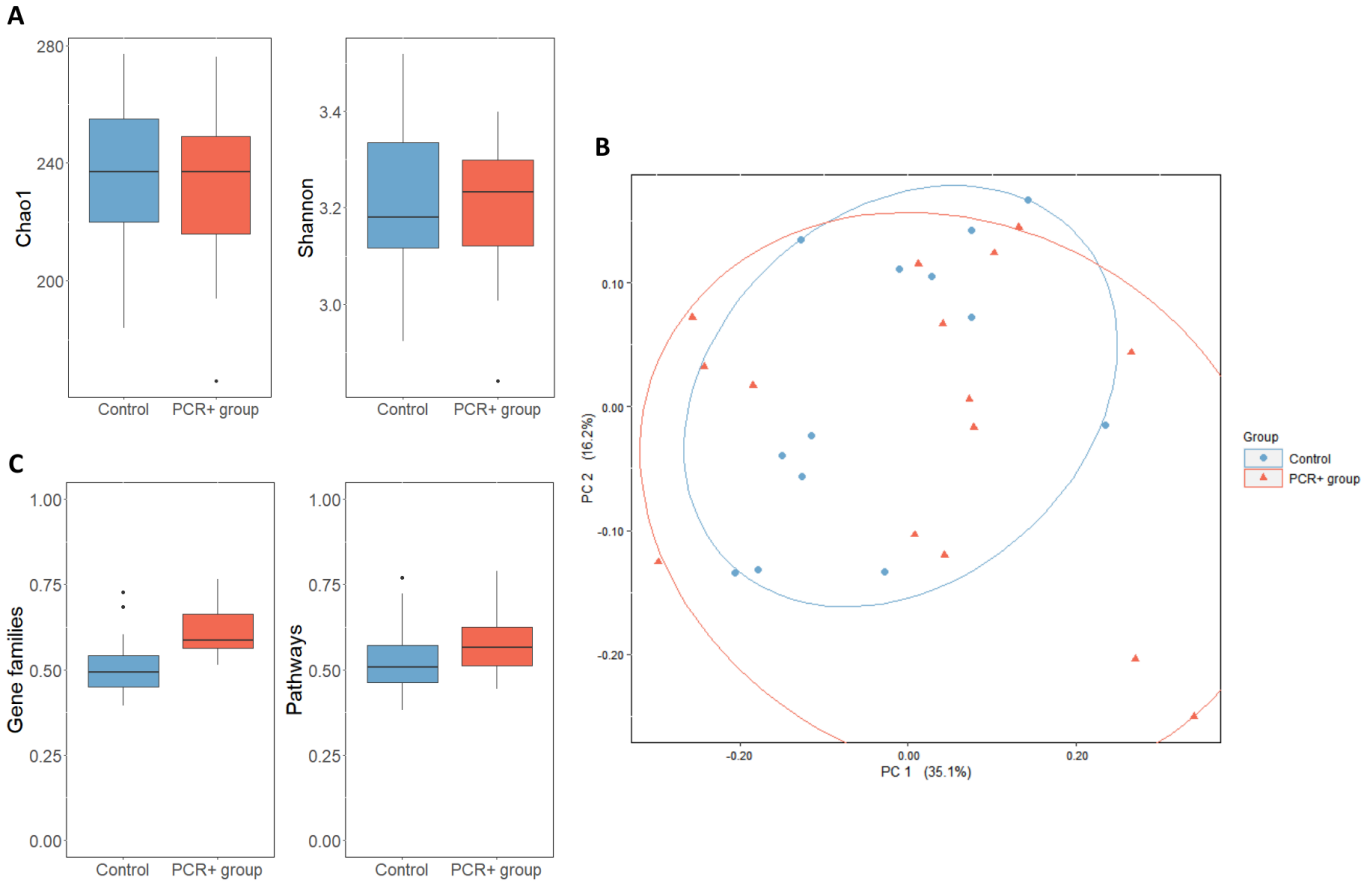
**(A)** α-diversity of control and PCR+ group, expressed as Chao1 and Shannon indexes. **(B)** Principal component analysis (PCoA) of microbial compositional data based on Bray-Curtis distances of control and PCR+ group samples. **(C)** Boxplot representing β-diversity of gene families and pathways, based on Bray-Curtis distances. Statistical differences were assessed through an ANOVA test for α- and β-diversity metrics, and a PERMANOVA test was applied for PCoA analysis.

After taxonomic assignment, 447 different species were detected in fecal samples. With regard to biodiversity, no differences were observed between groups in terms of Chao1 and Shannon indexes (**Figure 1A**). However, the PCoA analysis suggested a greater β-diversity for the PCR+ group, although this observation did not show statistical significance (PERMANOVA, R2 = 0.036, p-value = 0.422) (**Figure 1B**).

Statistical analysis for assessing differences in microbial profiles revealed changes in key taxa (**Tables 2**, **3**). With regard to phylum level, the PCR+ group showed higher proportions of *Verrucomicrobia* and *Synergistota* members than did controls, with an increase in *Akkermansiaceae* and *Synergistaceae* families in the PCR+ group and in *Veillonellaceae* members in the control group. Moreover, the proportions of *Roseburia*, *Slackia*, *Pediococcus*, *Sellimonas*, and *Acidaminococcus* genera, among others, were more abundant in the microbiome of the control group, as were those of *Blautia*, *Akkermansia*, *Eisenbergiella*, *Erysipelatoclostridium* and *Cloacibacillus* in the PCR+ group (**Table 2**). Recently, *Eisenbergiella* has been proposed to modulate the severity of COVID-19 by altering the ratio of dendritic cells, which play multiple critical roles in the infection process of SARS-CoV-2 (Li et al., 2024). Lastly, higher proportions of *Roseburia faecis*, *Slackia isoflavoniconvertens*, *Colinsella intestinalis*, and *Sellimonas intestinalis* were detected in the control group, along with some lactic acid bacteria (*Enterococcus faecalis*, *Leuconostoc gelidum*, *Lactobacillus plantarum*) and bifidobacteria (*Bifidobacterium catenulatum* and *Bifidobacterium dentium*). For the PCR+ group, higher levels of *Akkermansia muciniphila*, *Weissella confusa*, *Cloacibacillus porcorum*, and *Parabacteroides* sp. taxa were observed (**Table 3**).

**Table 2:** Relative abundance of significant taxa (p-adj < 0.05) at phylum, family, and genus levels, obtained by DESeq2, LefSE, and ANCOM statistical methods.

Taxonomic level	Taxa	Higher in	Control		PCR+ group	
			Mean	SD	Mean	SD
Phylum	Verrucomicrobia	PCR+ group	1.05%	3.35%	4.09%	7.98%
Phylum	Synergistota	PCR+ group	0.02%	0.06%	0.08%	0.21%
Family	*Veillonellaceae*	Control	0.95%	1.45%	0.27%	0.56%
Family	*Akkermansiaceae*	PCR+ group	1.05%	3.35%	4.09%	7.98%
Family	*Synergistaceae*	PCR+ group	0.02%	0.06%	0.08%	0.21%
Family	*Propionibacteriaceae*	PCR+ group	0.01%	0.02%	0.01%	0.04%
Genus	*Roseburia*	Control	3.83%	3.00%	2.49%	4.59%
Genus	*Slackia*	Control	0.95%	0.89%	0.36%	0.71%
Genus	*Pediococcus*	Control	0.84%	2.85%	0.00%	0.00%
Genus	*Sellimonas*	Control	0.22%	0.61%	0.03%	0.12%
Genus	*Acidaminococcus*	Control	0.19%	0.35%	0.02%	0.06%
Genus	*Veillonella*	Control	0.21%	0.56%	0.00%	0.01%
Genus	*Olsenella*	Control	0.05%	0.13%	0.00%	0.01%
Genus	*Blautia*	PCR+ group	4.09%	4.99%	7.99%	6.54%
Genus	*Akkermansia*	PCR+ group	1.05%	3.35%	4.09%	7.98%
Genus	*Eisenbergiella*	PCR+ group	0.08%	0.24%	0.71%	2.39%
Genus	*Erysipelatoclostridium*	PCR+ group	0.02%	0.07%	0.16%	0.40%
Genus	*Cloacibacillus*	PCR+ group	0.02%	0.06%	0.08%	0.20%
Genus	*Desulfovibrionaceae unclassified*	PCR+ group	0.01%	0.02%	0.05%	0.14%
Genus	*Weissella*	PCR+ group	0.02%	0.06%	0.02%	0.04%
Genus	*Coprobacter*	PCR+ group	0.00%	0.01%	0.02%	0.03%
Genus	*Propionibacterium*	PCR+ group	0.01%	0.02%	0.01%	0.04%

Only taxa with an abundance > 0.01% are shown.

**Table 3:** Relative abundance of significant taxa (p-adj < 0.05) at species level, obtained by DESeq2, LefSE, and ANCOM statistical methods.

Taxonomic level	Taxa	Higher in	Control		PCR+ group	
			Mean	SD	Mean	SD
Species	*Roseburia faecis*	Control	2.257%	2.073%	1.462%	3.154%
Species	*Slackia isoflavoniconvertens*	Control	0.947%	0.888%	0.359%	0.714%
Species	*Enterococcus faecalis*	Control	0.564%	1.551%	0.038%	0.102%
Species	*Collinsella intestinalis*	Control	0.278%	0.759%	0.007%	0.009%
Species	*Sellimonas intestinalis*	Control	0.223%	0.613%	0.031%	0.115%
Species	*Bacteroides stercoris*	Control	0.242%	0.724%	0.004%	0.010%
Species	*Leuconostoc gelidum*	Control	0.183%	0.564%	0.000%	0.001%
Species	*Bacteroides coprocola*	Control	0.123%	0.391%	0.019%	0.063%
Species	*Acidaminococcus intestini*	Control	0.157%	0.347%	0.013%	0.049%
Species	*Bifidobacterium catenulatum*	Control	0.102%	0.240%	0.048%	0.186%
Species	*Olsenella scatoligenes*	Control	0.048%	0.127%	0.002%	0.005%
Species	*Roseburia sp CAG 182*	Control	0.057%	0.175%	0.004%	0.010%
Species	*Bifidobacterium dentium*	Control	0.074%	0.231%	0.003%	0.008%
Species	*Actinomyces sp HPA0247*	Control	0.064%	0.229%	0.006%	0.020%
Species	*Eisenbergiella massiliensis*	Control	0.027%	0.065%	0.031%	0.120%
Species	*Lactobacillus plantarum*	Control	0.031%	0.074%	0.001%	0.004%
Species	*Veillonella atypica*	Control	0.032%	0.064%	0.001%	0.003%
Species	*Eubacterium sp OM08 24*	Control	0.023%	0.081%	0.009%	0.018%
Species	*Ruminococcus obeum CAG 39*	Control	0.011%	0.032%	0.005%	0.019%
Species	*Clostridium lavalense*	Control	0.011%	0.021%	0.002%	0.004%
Species	*Clostridium sp CAG 253*	Control	0.012%	0.027%	0.000%	0.000%
Species	*Veillonella dispar*	Control	0.013%	0.023%	0.001%	0.003%
Species	*Weissella cibaria*	Control	0.017%	0.060%	0.003%	0.008%
Species	*Streptococcus vestibularis*	Control	0.009%	0.020%	0.001%	0.002%
Species	*Rothia mucilaginosa*	Control	0.006%	0.010%	0.002%	0.008%
Species	*Akkermansia muciniphila*	PCR+ group	1.050%	3.350%	4.088%	7.976%
Species	*Dorea longicatena*	PCR+ group	0.671%	1.868%	1.325%	1.663%
Species	*Dorea formicigenerans*	PCR+ group	0.426%	0.676%	0.968%	1.260%
Species	*Eisenbergiella tayi*	PCR+ group	0.056%	0.180%	0.680%	2.395%
Species	*Clostridium innocuum*	PCR+ group	0.004%	0.011%	0.133%	0.398%
Species	*Bacteroides clarus*	PCR+ group	0.012%	0.023%	0.098%	0.243%
Species	*Parabacteroides goldsteinii*	PCR+ group	0.055%	0.147%	0.093%	0.238%
Species	*Alistipes indistinctus*	PCR+ group	0.015%	0.026%	0.149%	0.278%
Species	*Parabacteroides johnsonii*	PCR+ group	0.001%	0.004%	0.063%	0.135%
Species	*Lactococcus lactis*	PCR+ group	0.023%	0.069%	0.028%	0.071%
Species	*Cloacibacillus porcorum*	PCR+ group	0.004%	0.014%	0.072%	0.178%
Species	*Bacteroides massiliensis*	PCR+ group	0.031%	0.103%	0.044%	0.070%
Species	*Erysipelatoclostridium ramosum*	PCR+ group	0.020%	0.071%	0.016%	0.052%
Species	*Weissella confusa*	PCR+ group	0.000%	0.000%	0.018%	0.034%
Species	*Clostridium spiroforme*	PCR+ group	0.000%	0.000%	0.012%	0.031%
Species	*Streptococcus mitis*	PCR+ group	0.001%	0.003%	0.008%	0.012%
Species	*Leuconostoc mesenteroides*	PCR+ group	0.000%	0.001%	0.004%	0.009%
Species	*Gemella haemolysans*	PCR+ group	0.001%	0.002%	0.001%	0.003%

#### 3.2.2 Differences in gut microbial functionality and metabolism in persistent positive RT-PCR condition

HUMAnN 3.0 functional assignment resulted in 4,474,633 different gene families and 14,083 metabolic pathways. The PCR+ group showed greater β-diversity of gene families and pathways, although only the gene families were statistically significant (p-value = 0.01) (**Figure 1C**). The differential abundance analysis revealed that the functionality of the gut microbiome changed after viral persistence for more than 4 weeks. The fecal microbiome of the PCR+ group was enriched in functional pathways involved in the biosynthesis of amino acids (L-arginine, L-methionine, L-isoleucine, L-lysine, and chorismate), nucleotides (guanosine ribonucleotides, UMP, and 5-aminoimidazole ribonucleotide), and carbohydrates (mannose, rhamnose, glycero-manno-heptose). In contrast, some pathways related to peptidoglycan and amino acids biosynthesis and glycolysis were highlighted in the control group (**Supplementary Table 4**).

We next investigated whether PCR+ altered stool SCFA profiles in the study participants, as a proxy measure of microbiome alteration. When comparing the changes in SCFA levels between groups, there was no significant difference between them. However, we found that most SCFAs exhibited a similar pattern, with lower levels of total SCFAs in individuals with PCR+ compared with the controls, mainly propionic and butyric acids, and to a lesser extent of isobutyric, isovaleric, and valeric acids (**Supplementary Table 5**).

### 3.3 Integrative analysis through a multi-omics approach

**Figure 2:**
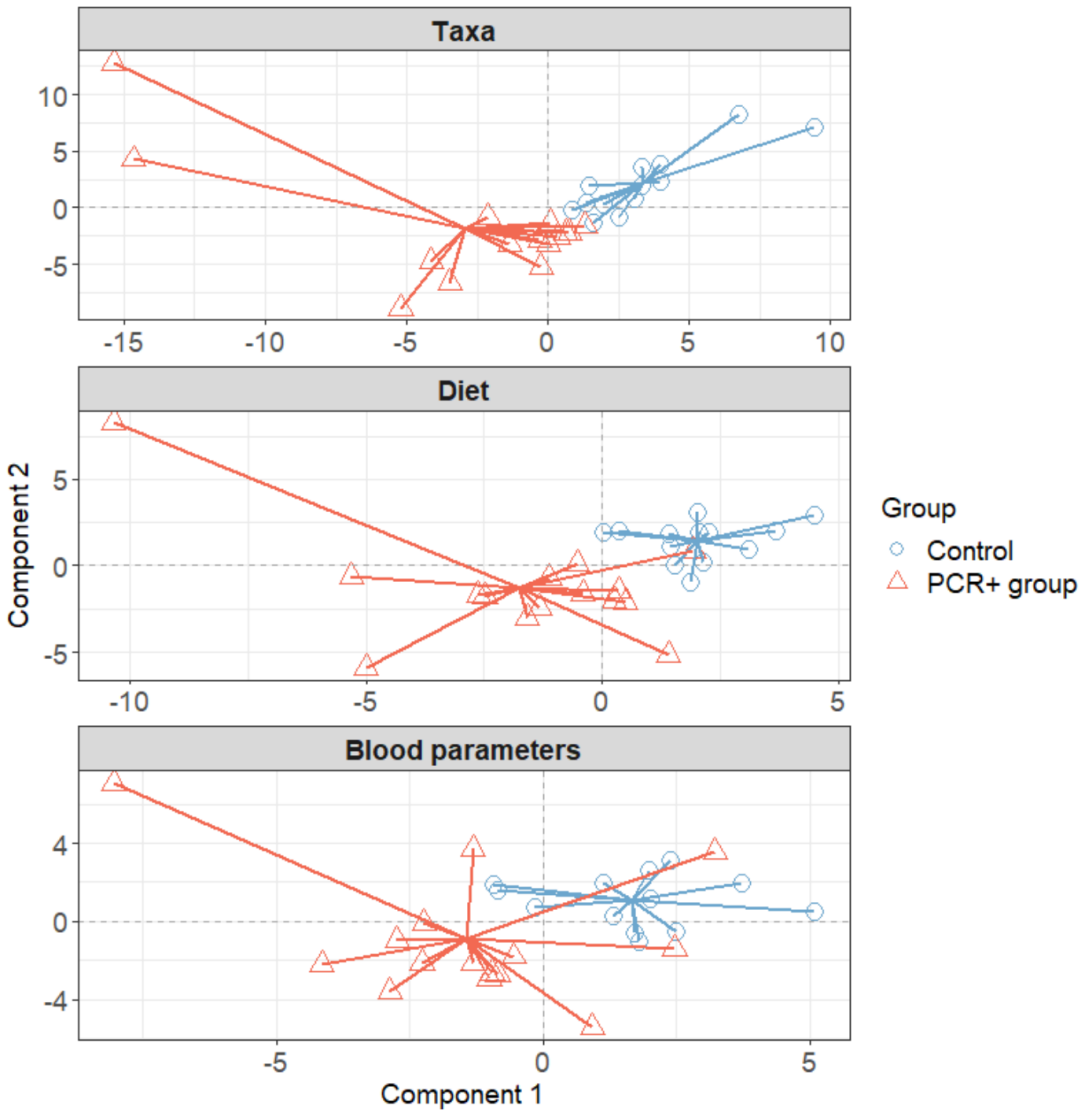
Plots in which samples are grouped according to two-component score for each dataset, obtained using the DIABLO method of the mixOmics library (R software). Initially all datasets were included: taxa, functional profiling, diet, blood parameters, immune response, and SCFAs. To improve the model, only datasets with a weight > 0.5 were considered (taxa, blood parameters, and diet).

**Figure 3:**
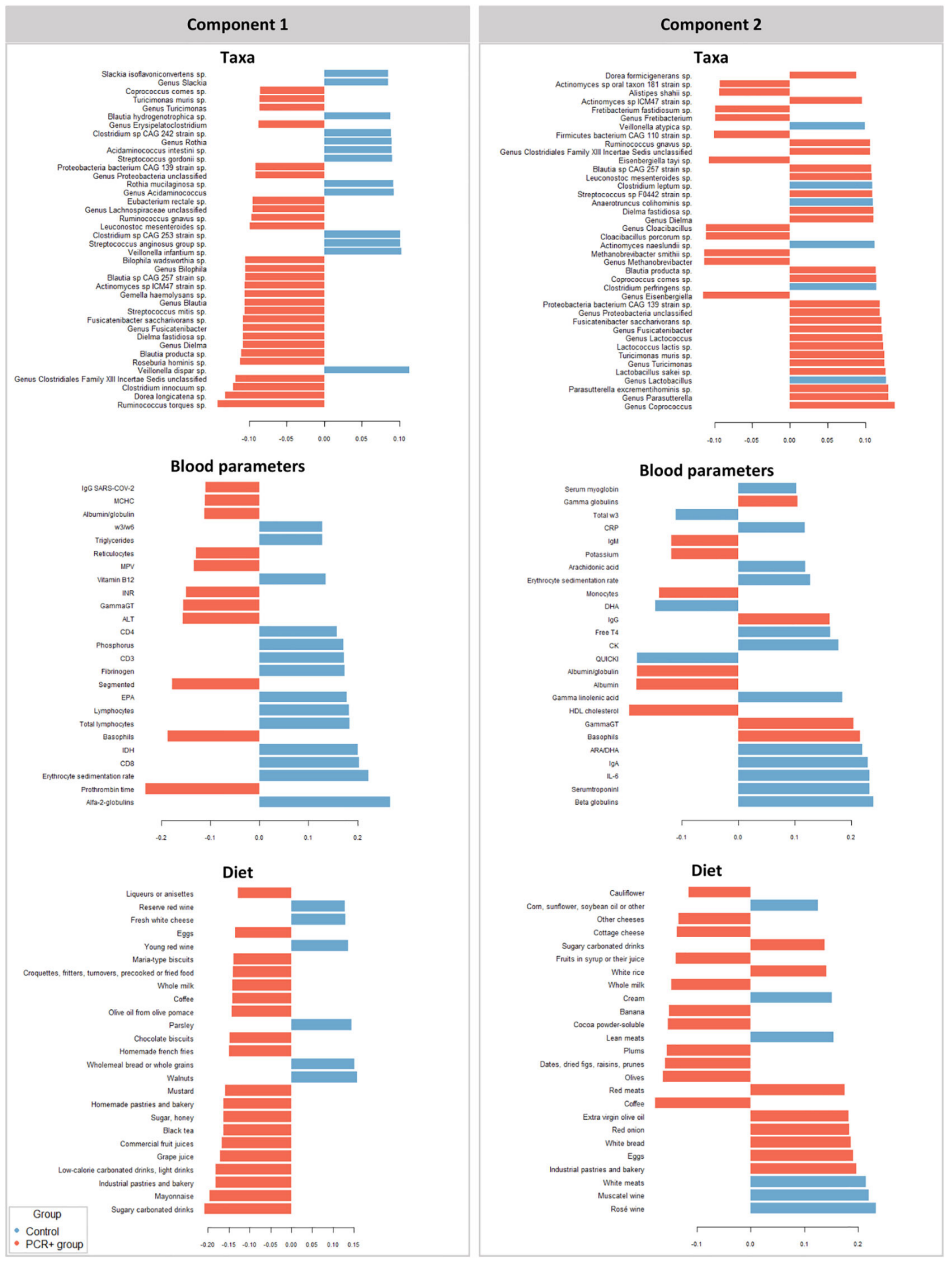
Variables that contribute the most to the differences observed between groups in components 1 and 2 in the different datasets studied (taxa, diet, and blood parameters). The first 40 contributing variables are shown in taxa and 25 for blood parameters and diet datasets.

We further integrated the results from metagenomics, metabolites, and metadata using a multivariate approach using the DIABLO method from the mixOmics package (Rohart et al., 2017) to identify features across the different datasets that could discriminate between groups. To improve the DIABLO model, only datasets with component weights greater than 0.50 were included. A total of 510 variables among taxa, dietary features, and blood parameters were considered. The results showed differences between the study groups for all the datasets analyzed (**Figure 2**). The main taxa contributors to components 1 and 2 of the model were in line with previous statistical analysis (**Figure 3**). At the genus level, *Blautia*, *Eisenbergiella*, and *Cloacibacillus* again showed their important involvement in the microbiome of the PCR+ group, and primarily *Slackia* and *Acidaminococcus* in the control group. Moreover, species of *Dorea*, *Gemella haemolysans*, *Streptococcus mitis*, *Leuconostoc mesenteroides*, *Cloacibacillus porcorum*, and *Lactococcus lactis* were highlighted in the PCR+ group, and *Acidaminococcus intestini*, *Slackia isoflavoconvertans*, some *Veillonella* spp., and *Rothia mucilaginosa* in the control group. With regard to blood parameters, some markers were more represented in the PCR+ group, including higher prothrombin time, segmented neutrophils and basophils, gamma-glutamyl transferase (gammaGT), alanine aminotransferase (ALT), SARS-CoV-2 specific and general immunoglobulin G (IgG), and immunoglobulin M (IgM). Interestingly, the control group showed higher levels of important nutrients such as vitamin B12, arachidonic acid (ARA), docosahexaenoic acid (DHA), eicosapentanoic acid (EPA), omega-3 fatty acids (ω3), as well as immunoglobulin A (IgA). Furthermore, the PCR+ group was associated with a diet rich in nonalcoholic and sugar drinks (sugary or low-calorie/light carbonated drinks, juices, and others), red meat, sugar/honey, bakery products and pastries (biscuits, homemade and industrial pastries and bread, and others), and miscellaneous foods (fried or precooked food, sauces, and others).

**Figure 4:**
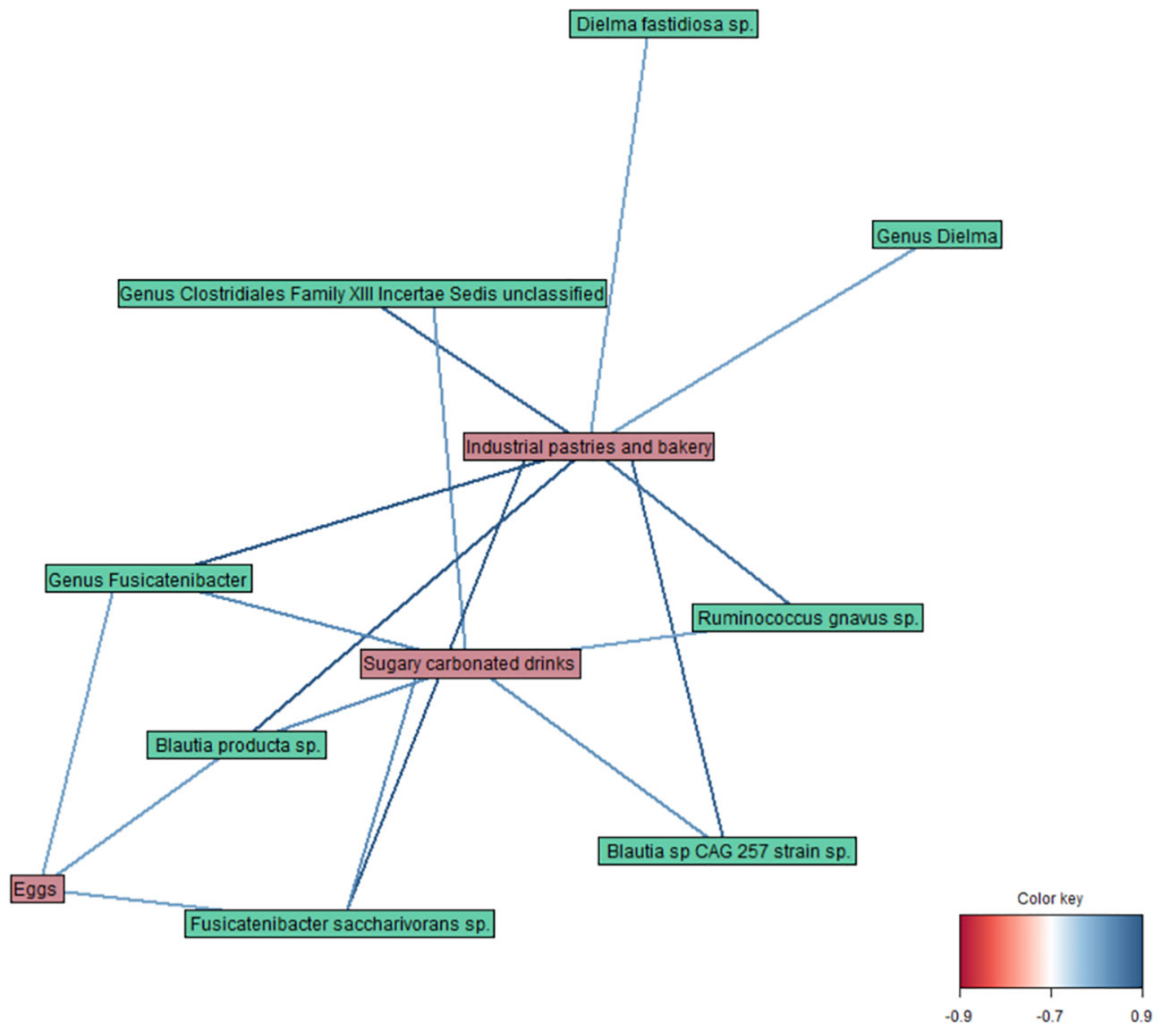
Network plot representing the correlations between variables obtained by the DIABLO method in taxa, diet, and blood parameter datasets (r = 0.8).

Finally, the DIABLO method (r = 0.8) revealed correlations between different variables of the datasets (**Figure 4**), such as a strong positive link of *Fusicatenibacter saccharivorans* and *Blautia producta* with industrial pastries and bakery products, and a weaker one with sugary carbonated drinks and egg consumption. Moreover, *Ruminococcus gnavus*, *Blautia* sp CAG 257, and members of the genus unclassified *Clostridiales* Family XIII Incertae Sedis were also correlated with industrial pastries and bakery products and sugary carbonated drinks; and *Dielma fastidiosa* with industrial pastries and bakery products.

## 4 Discussion

COVID-19 was able to affect gut composition and biodiversity by shifting it towards a state of dysbiosis (Hazan et al., 2022; Schult et al., 2022), an imbalance that could be maintained even months after the recovery (Tian et al., 2021; Ferreira-Junior et al., 2022). Patients with a dysbiotic gut microbiota have a higher risk of developing a more severe form of COVID-19, and key microbial markers associated with this disease has been suggested (Mancabelli et al., 2022). Moreover, gut dysbiosis has been linked to numerous health problems in long COVID-19/post-acute COVID-19 patients (Raj et al., 2024). Therefore, understanding how the gut microbiota may be altered and functionally influenced by SARS-CoV-2 and its pathogenic toxins is a major focus of current research. In our study, both the PCR+ and the control COVID-19 groups showed a gut dysbiosis compared to what has previously been described as a “healthy” state (Manor et al., 2020), which appears to be sustained over time following infection.

First, in the analysis of gut microbial diversity and composition, the PCR+ group showed remarkable differences in specific taxa, characterized by enrichment of potential pathogenic bacteria, such as *Erysipelatoclostridium*, *Cloacibacillus*, and *Weissella*. Some strains of *Erysipelatoclostridium ramosum* are capable of increasing host susceptibility to opportunistic bacterial invasion via translocation across the intestinal mucosa (Milosavljevic et al., 2021), whereas *Cloacibacillus porcorum* and *Weissella confusa* have been associated with different diseases and bacteremia (Domingo et al., 2015; Kamboj et al., 2015; Kodaka et al., 2022). Additionally, *Blautia* and *Akkermasia* were also increased in PCR+ subjects, although their contribution to health is controversial. For instance, it has been stated that increased levels of *Akkermansia muciniphila* may aggravate some metabolic and inflammatory pathologies (Xue et al., 2023). Some lactic acid bacteria were also highlighted in the PCR+ group, which may be favored by the proinflammatory environment resulting from the dysbiotic state and related acidic conditions (Rajamäki et al., 2013). SARS-CoV-2 seems to remain longer in feces than in the respiratory tract (Wu et al., 2020; Okita et al., 2022; Natarajan et al., 2022), which is not necessarily related to gastrointestinal symptoms and disease severity (Gaebler et al., 2021; Maeda et al., 2022). A longer duration of positive RT-PCR in nasal samples could cause a prolonged presence of the virus in the gut, which could extend the associated gut dysbiosis over time or hinder the reestablishment of gut microbiome homeostasis.

Conversely, the control group showed higher levels of commensals and taxa related to positive health effects, such as *Roseburia* and *Slackia*. *Roseburia* spp. constitute important denizens of the human gut microbiome that ferment complex polysaccharides to butyrate, with known beneficial effects on gut health (Hillman et al., 2020). In addition, *Slackia* members perform key metabolic functions for host homeostasis (Kim et al., 2020). Moreover, increased levels of some *Bifidobacterium* and *Lactobacillus* species in controls, also well-known for their health benefits and potential probiotic effects, should be highlighted (Chen et al., 2021; Rastogi and Singh, 2022). However, some opportunistic pathogen bacteria, like *Veillonella dispar* and *Rothia mucilaginosa* were increased in controls, in agreement with previous studies in COVID-19 patients (Tian et al., 2021), suggesting a long-term impact on the gut bacteriome. *Rothia mucilaginosa* is a normal component of the oral and respiratory tract microbiome, but the fecal enrichment of specific oral taxa, linked with increased oral–fecal microbial transmission, has frequently been regarded as a hallmark of disease (Schmidt et al., 2019). In our study, the observed effect might be attributed to the fact that both study groups experienced COVID-19, therefore the control group may not have fully restored their microbiota in post-infection recovery.

We further explored functional and metabolic differences in gut microbiome, revealing an enrichment in the PCR+ group of functional pathways involved in nucleotide, amino acid, and carbohydrate biosynthesis, suggesting a dysbiotic microbiome trying to recover functional balance. Zuo et al. (2021) stated that increased nucleotide and amino acid biosynthesis in gut microbiome functionality indicates potential enhanced production of bacterial cellular building blocks for macromolecules, and carbohydrates are structural components of bacterial cell wall (Zuo et al., 2021). The increased mannose biosynthesis could be linked to glycerol-manno-heptose synthesis, which is also a key compound of the cell wall and core region of lipopolysaccharide (LPS) of Gram-negative bacteria (Kneidinger et al., 2001). Rhamnose is also part of Gram-positive bacteria cell walls, being critical in cell wall structure, biogenesis, and pathogenesis (Mistou et al., 2016). Therefore, these functions, together with the catabolism of uncommon carbon sources such as myo-inositol, could be involved in promoting microbial growth, but they could also contribute to a greater harmful effect on the host’s health (Caputo et al., 2020). SCFA production in this group was also affected, suggesting that the PCR+ condition may also be associated with impaired capacity of the gut microbiome to produce these beneficial metabolites, in line with previously reported effects, including severe COVID-19 patients (Zhang et al., 2022). Taken together, these findings suggest that individuals who suffered from the COVID-19 with persistent positive RT-PCR have a different gut microbiome profile compared to those who tested positive for a normal length of time. Further studies are needed to determine the clinical significance and mechanisms of these differences.

It is noteworthy that the integration of the different datasets using mixOmics also showed differences between the PCR+ and control groups in all the datasets studied. When looking at the variables that contribute the most to the observed differences, the results of microbial taxa supported those previously observed in the differential abundance analysis. In addition to those previously mentioned, the integrative analysis also revealed other potential pathogenic taxa in the PCR+ group*. Ruminococcus gnavus* and *Ruminococcus torques* have been associated with inflammatory bowel disease and diabetes (Crost et al., 2023). Notably, higher levels of *Ruminococcus gnavus* have been reported in people with long COVID-19 compared with non-COVID-19 controls, with gut dysbiosis lasting 14 months (Davis et al., 2023). Moreover, *Streptococcus mitis* and *Fretibacterium fastidiosum* are part of the oral microbiota (Mitchell, 2011; Vartoukian et al., 2013), so their detection in the gut could be linked to imbalanced oral–gut translocation, related to different disorders, such as inflammatory bowel disease, among others (Abdelbary et al., 2022). Conversely, health-related *Lactobacillus* members were more abundant in controls. Given the above, this analysis confirms that the PCR+ group harbors higher levels of bacteria linked to negative health effects than controls, thus contributing to a greater dysbiotic state.

The PCR+ group was also associated with an animal protein-rich diet, and processed, precooked, oily, and high-sugar-content food. Previous research has linked a plant-based food diet or a high adherence to a Mediterranean diet with lower infection and mortality rates caused by SARS-CoV-2 (Vu et al., 2021; Perez-Araluce et al., 2022) and a reduced risk of contracting severe COVID-19 (Kim et al., 2021). Moreover, individuals with COVID-19 reported lower intakes of water, plant protein, seeds, legumes, garlic, dietary fiber, and various vitamins and minerals, as well as following diets that were higher on the dietary inflammation index (Jagielski et al., 2022). Indeed, plant-based food consumption may help improve dysbiotic gut microbiota in COVID-19 and long-COVID patients, potentially aiding disease management (Li et al., 2023), due to its high content of bioactive compounds, with anti-inflammatory, antibacterial, and antiviral properties (Campbell, 2023). Therefore, our findings suggest a potential protective role of healthy dietary patterns, and that an unhealthy diet could also increase the risk of developing the persistent positive RT-PCR condition.

Additionally, evidence suggests that the gut microbiome plays a key role in tackling viral infections by modulating antiviral innate and adaptive leucocyte function in the host immune system (Rossini et al., 2024). The cellular and humoral immune analysis confirmed that both groups show efficient immunity. Moreover, our results highlighted a correlation between persistent positive RT-PCR and basophil and segmented neutrophil levels, responsible for coordinating the adaptive immune response and crucial in the first line of cell-mediated defense against SARS-CoV-2. Increased neutrophil counts have been detected in blood as a feature of COVID-19, particularly in severe cases (Reusch et al., 2021). Moreover, the number of basophils is lower during acute disease than in the recovery phase, as it is a determinant of the antibody response to the virus (Murdaca et al., 2021). In the case of persistent positive RT-PCR, as the majority of the participants were asymptomatic or had mild symptoms, basophils and neutrophils could have helped in dealing with the prolonged shedding of the virus, and could have acted as antigen-presenting cells boosting humoral immune responses. General and SARS-CoV-2 IgG and IgM were correlated to this group, which could be a consequence of basophil actuation together with a longer exposure of the immune system to the virus. In addition, some liver injury markers like gamma GT, ALT, and prothrombin time were increased in the PCR+ group, probably due to the prolonged presence of the virus in the organism and a possible liver infection (Zhao et al., 2023). On the other hand, some parameters that were increased in controls have been associated with a better prognosis after SARS-CoV-2 infection. Vitamin B12 could act as a potential inhibitor against the virus and help balance immune responses during viral infections (Batista et al., 2022). Moreover, higher omega-3 levels, and specifically DHA, have been associated with a lower risk of testing positive for infection with SARS-CoV-2, hospitalization, and mortality (Calder, 2023). ARA could act as an endogenous antiviral compound and its deficiency could increase the susceptibility to COVID-19 (Hoxha, 2020). In addition, IgA controls the initial neutralizing antibody response to SARS-CoV-2 (Esmat et al., 2024); therefore, higher levels of IgA in the blood could be involved in neutralizing the virus and reducing the persistence of positive tests.

Finally, among all the datasets studied in the integrative analysis, relevant correlations were observed between some microbial taxa and food/diet components, highlighting their potential role in the PCR+ condition. Notably, animal protein (eggs) and oily and sugary food (pastries and soft drinks) were associated with specific taxa. Highlighting the relationships between diet and gut microbiome, and their repercussions in microbiome-host metabolic interactions is one of the main current challenges in numerous aspects of human biology, including metabolic health to infections and immune functions (Mancabelli et al., 2024). Previous studies have associated animal-derived food and a high-sugar high-fat diet with higher abundances of *Ruminococcus* spp. *Ruminococcus gnavus* and *Blautia*, among other taxa (Bolte et al., 2021; Lee et al., 2023). Moreover, *Fusicatenibacter saccharivorans* proportions seem to increase after the intake of processed food (Matsuyama et al., 2019), and a moderately high intake of protein has been linked to a decrease in *Dielma* and *Dielma fastidiosa* levels (Cuevas-Sierra et al., 2021). A possible explanation for this could be that the presence of SARS-CoV-2 in the gut, coupled to a diet with a high intake of animal protein, sugar, and saturated fats, could favor certain microbial taxa, disrupting the balance of microbial communities and potentially leading to a dysbiotic state.

This study has several limitations, primarily due to the relatively small sample size and the retrospective nature of the investigation. Furthermore, the collection of samples was occasionally delayed because of technical difficulties and local restrictions. A further limitation is that we did not determine fecal shedding of SARS-CoV-2 RNA or the presence of live viruses in the gut, as this could also influence the observed effects on the microbiome. For safety and practical reasons, it was necessary to process the fecal samples for virus inactivation. Nevertheless, this study offers new insights and corroborates existing knowledge. In particular, we shed new light on complex SARS-CoV-2-microbiome interactions highlighting the overlap with host conditions. Our findings illustrate the varying impacts of SARS-CoV-2 and COVID-19 infection on the intestinal microbiome and its metabolic functionality, contingent on the duration of viral persistence within the organism. Gut microbiome dysbiosis may persist for several months in individuals who have persistently positive RT-PCR results. Furthermore, our results indicated that certain blood markers and dietary habits are associated with prolonged positivity, suggesting the potential value of appropriate nutritional interventions as a part of a comprehensive care strategy for patients recovering from COVID-19. All in all, this study stands out as pioneer to integrate different omics approaches and datasets in a PCR persistent SARS-CoV-2 cohort and provides valuable data for deepening the understanding of the functional contribution of gut microbiota in infection and human health. Data presented here, when placed in the context of preliminary work, underscore the necessity of incorporating the gut microbiota into clinical trials to elucidate the biological mechanisms underlying disease, including viral persistence and the impact of disease on multiple organ systems.

## Data Availability

The data presented in the study are deposited in the Sequence Read Archive (SRA) repository, accession number PRJNA1099539.
