# Supplementary material for: Gut microbiome and clinical and lifestyle host factors associated with recurrent positive RT-PCR for SARS-CoV-2
Source: Front Cell Infect Microbiol. 2024 Dec 18;14:1494193. doi: 10.3389/fcimb.2024.1494193 (PMC11688222; doi:10.3389/fcimb.2024.1494193)
Supplement: Supplementary file 1 [file DataSheet1.pdf]

Supplementary Material

1 Supplementary Tables

Supplementary Table 1. Parameters of blood analysis in control and PCR+ groups. An ANOVA test was applied for parametric variables, and the Kruskal-Wallis test was implemented to assess differences between nonparametric ones. p-values were adjusted using the BH correction. No statistical differences were found between groups (p-adj < 0.05).

Blood parameter	Control	PCR+ group	p-value	p-adj
Leukocytes (10^3/µl)	5.49 ± 1.59	5.35 ± 1.14	0.79	0.90
Red blood cells (10^6/µl)	4.92 ± 0.41	5.01 ± 0.50	0.62	0.89
Hemoglobin (g/dl)	14.59 ± 1.46	15.06 ± 1.15	0.35	0.86
Hematocrit (%)	44.31 ± 3.51	45.43 ± 3.42	0.40	0.86
MCV (fl)	90.18 ± 5.90	90.98 ± 5.07	0.70	0.89
MCH (pg)	29.67 ± 2.21	30.15 ± 1.57	0.89	0.95
MCHC (g/l)	32.89 ± 1.08	33.15 ± 0.54	0.66	0.92
IDH (%)	13.26 ± 0.49	12.95 ± 0.43	0.09	0.55
Platelets (10^3/µl)	254.00 ± 46.51	241.33 ± 50.28	0.50	0.86
MPV (fl)	9.11 ± 1.05	9.58 ± 0.96	0.22	0.79
Reticulocytes (%)	1.23 ± 0.39	1.47 ± 0.49	0.18	0.66
Lymphocytes (%)	32.75 ± 9.03	27.75 ± 6.62	0.10	0.55
Monocytes (%)	6.52 ± 1.84	8.43 ± 8.56	0.80	0.92
Segmented (%)	58.02 ± 10.26	63.54 ± 7.23	0.11	0.55
Eosinophils (%)	2.12 ± 0.83	2.03 ± 1.31	0.39	0.92
Basophils (%)	0.59 ± 0.20	0.79 ± 0.91	0.90	0.95
Erythrocyte sedimentation rate (mm/h)	18.15 ± 14.95	7.47 ± 4.82	0.05	0.92
Prothrombin time (sg)	12.07 ± 0.74	12.46 ± 0.50	0.11	0.55
QUICKI (%)	97.23 ± 4.17	96.87 ± 4.78	0.94	0.96
INR	0.98 ± 0.06	1.00 ± 0.05	0.45	0.92
Fibrinogen (mg/dl)	344.85 ± 87.86	289.27 ± 61.33	0.05	0.92
D-Dimer (µg/l)	286.92 ± 157.87	257.33 ± 128.42	0.59	0.87
Glucose (mg/dl)	91.00 ± 19.45	89.07 ± 4.93	0.42	0.92
Urea (mg/dl)	33.31 ± 8.62	35.20 ± 11.20	0.71	0.92
NUN (mg/dl)	15.54 ± 4.02	16.42 ± 5.22	0.71	0.92
Creatinine (mg/dl)	0.77 ± 0.19	0.82 ± 0.22	0.52	0.86
Total bilirubin (mg/dl)	0.75 ± 0.30	0.94 ± 0.68	0.32	0.92
Uric acid (mg/dl)	7.39 ± 9.24	4.79 ± 1.28	0.46	0.92
Iron (µg/dl)	88.92 ± 30.08	102.00 ± 47.36	0.64	0.92
Calcium (mg/dl)	9.49 ± 0.48	9.53 ± 0.39	0.81	0.90
Phosphorus (mg/dl)	3.28 ± 0.47	3.06 ± 0.55	0.28	0.86
AST (UI/l)	20.77 ± 4.11	20.40 ± 4.82	0.83	0.90
ALT (UI/l)	18.69 ± 7.11	21.73 ± 9.84	0.49	0.92
Alkaline phosphatase (UI/l)	74.23 ± 16.92	70.07 ± 19.85	0.56	0.87
GammaGT (UI/l)	16.69 ± 6.32	18.93 ± 10.00	0.78	0.92
LDH (UI/l)	299.15 ± 38.23	304.53 ± 37.68	0.71	0.89
Amylase (UI/l)	57.15 ± 20.59	57.73 ± 22.86	1.00	1.00
CK (UI/l)	114.08 ± 69.02	95.93 ± 41.33	0.71	0.92
Sodium (mEq/l)	141.15 ± 1.28	141.53 ± 1.51	0.48	0.86
Potassium (mEq/l)	4.52 ± 0.48	4.68 ± 0.48	0.38	0.86
Total cholesterol (mg/dl)	191.23 ± 34.62	190.00 ± 28.47	0.92	0.96
Triglycerides (mg/dl)	87.54 ± 42.47	72.60 ± 23.68	0.49	0.92
HDL cholesterol (mg/dl)	59.00 ± 17.64	63.8 ± 15.22	0.45	0.86
LDL cholesterol (mg/dl)	114.62 ± 26.8	111.87 ± 24.72	0.75	0.92

Total protein (g/dl)	6.89 ± 0.46	6.95 ± 0.52	0.75	0.89
Albumin (%)	60.25 ± 2.11	61.95 ± 3.04	0.10	0.55
Alfa-1-globulins (%)	3.91 ± 0.64	3.83 ± 0.64	0.76	0.89
Alfa-2-globulins (%)	10.25 ± 1.43	8.93 ± 1.30	0.02	0.55
Beta globulins (%)	10.88 ± 0.97	10.51 ± 1.14	0.37	0.86
Gamma globulins (%)	14.71 ± 1.63	14.78 ± 2.50	0.93	0.96
Albumin/globulin	1.52 ± 0.14	1.64 ± 0.20	0.09	0.55
Ferritin (ng/ml)	110.32 ± 128.68	105.83 ± 96.46	0.84	0.93
C reactive protein (mg/dl)	0.25 ± 0.45	0.24 ± 0.47	0.21	0.92
TSH (µUI/ml)	2.20 ± 0.71	1.85 ± 1.09	0.12	0.92
Free T4 (ng/dl)	1.26 ± 0.11	1.19 ± 0.20	0.26	0.92
Serum myoglobin (ng/ml)	41.26 ± 18.6	39.27 ± 8.55	0.59	0.92
Serum troponin I (ng/l)	2.58 ± 2.22	1.90 ± 1.44	0.47	0.92
ProBNP (pg/ml)	48.85 ± 26.25	58.60 ± 35.86	0.36	0.92
Procalcitonin (ng/ml)	0.01 ± 0.00	0.01 ± 0.01	0.61	0.92
IgG (mg/dl)	1060 ± 121.93	1064.8 ± 213.59	0.94	0.94
IgA (mg/dl)	227.92 ± 114.68	209.6 ± 134.36	0.60	0.92
IgM (mg/dl)	109.15 ± 49.23	109.53 ± 61.08	0.76	0.92
IgG SARS-CoV-2 (U/dl)	153.5 ± 263.79	218.45 ± 500.02	0.37	0.92
IgM SARS-CoV-2 (U/dl)	0.36 ± 0.40	0.38 ± 0.23	0.50	0.92
Total lymphocytes (cells/ml)	1792.69 ± 323.19	1494.73 ± 515.03	0.08	0.55
Lymphocyte CD3 (%)	73.01 ± 4.98	71.44 ± 7.50	0.53	0.86
CD3 (cells/ml)	1310.31 ± 262.76	1077.27 ± 436.11	0.11	0.55
CD3/CD4 (%)	44.28 ± 6.09	43.62 ± 5.48	0.77	0.89
CD4 (cells/ml)	795.23 ± 197.11	662.93 ± 275.17	0.16	0.66
CD3/CD8 (%)	26.40 ± 5.74	24.96 ± 7.24	0.57	0.87
CD8 (cells/ml)	471.62 ± 127.96	369.67 ± 157.25	0.07	0.55
CD4/CD8 (%)	1.80 ± 0.66	1.91 ± 0.66	0.65	0.89
IL-6 (pg/ml)	3.64 ± 2.93	2.92 ± 2.26	0.11	0.92
Vitamin D (ng/ml)	29.76 ± 17.58	28.06 ± 10.34	0.73	0.92
Vitamin B12 (pg/ml)	385.31 ± 98.24	333.53 ± 79.15	0.13	0.60
DHA (µmol/l)	343.62 ± 86.49	329.67 ± 112.34	0.72	0.89
EPA (µmol/l)	78.15 ± 38.66	57.33 ± 25.20	0.11	0.92
Total w3 (µmol/l)	421.77 ± 114.73	387.07 ± 129.31	0.46	0.86
Arachidonic acid (µmol/l)	535.38 ± 118.04	478.47 ± 132.29	0.24	0.80
Linoleic acid (µmol/l)	1680.39 ± 324.46	1745.33 ± 439.51	0.66	0.89
Gamma linolenic acid (µmol/l)	16.92 ± 6.40	14.93 ± 8.95	0.51	0.86
Eicosadienoic acid (µmol/l)	13.77 ± 6.30	12.40 ± 4.12	0.50	0.86
Eicosatrienoic acid (µmol/l)	178.31 ± 53.28	164.67 ± 64.86	0.20	0.92
Total w6 (µmol/l)	2424.77 ± 421.49	2415.67 ± 555.64	0.96	0.96
ARA/EPA	8.23 ± 3.59	9.20 ± 2.68	0.42	0.86
ARA/DHA	1.69 ± 0.63	1.60 ± 0.83	0.54	0.92
w3/w6	0.18 ± 0.05	0.16 ± 0.04	0.39	0.86

Supplementary Table 2. Daily consumption of food groups (g/day) estimated by FFQ questionnaire for the control and PCR+ groups. An ANOVA test was applied for parametric variables, and the Kruskal-Wallis test was implemented to assess differences between nonparametric ones. p-values were adjusted using the BH correction. Data are expressed as mean ± SD. No statistical differences were found between groups (p-adj < 0.05).

Food group	Control	PCR+ group	p-value	p-adj
Pastries, cakes, and sweets	25.78 ± 21.65	51.69 ± 59.70	0.123	0.792
Fruits	665.23 ± 325.89	580.47 ± 599.16	0.147	0.792
Dairy products	363.02 ± 186.34	287.62 ± 183.26	0.291	0.525
Miscellaneous (precooked foods and others)	26.17 ± 20.36	38.42 ± 35.69	0.300	0.792
Nonalcoholic drinks	1170.43 ± 386.08	1382.90 ± 715.20	0.348	0.525
Alcoholic drinks	100.20 ± 91.82	77.84 ± 91.17	0.433	0.792
Total drinks	1271.17 ± 393.12	1461.67 ± 701.31	0.394	0.525
Fats	28.27 ± 18.95	25.12 ± 19.88	0.504	0.792
Legumes and cereals	208.62 ± 129.29	173.71 ± 108.61	0.504	0.792
Olive oil and others	47.74 ± 25.14	46.89 ± 26.56	0.627	0.859
Vegetables	611.47 ± 353.99	568.56 ± 308.44	0.730	0.859
Eggs	22.77 ± 11.42	25.40 ± 35.73	0.368	0.792
Meat and meat products	120.7 ± 63.47	131.51 ± 103.26	0.982	0.982
Fish and seafood	74.47 ± 35.15	70.53 ± 38.63	0.781	0.859
Total protein food	216.32 ± 93.76	227.45 ± 138.30	0.808	0.808

FFQ consumption frequencies for the 118 different items were adjusted to daily frequencies, ranging from 0 (no consumption) to 7 (consumption more than 6 times a day), and multiplied by the grams equivalent to a standard portion of each item, resulting in the daily consumption value in grams. The intake of different food groups was determined by aggregating the daily consumption (g/day) of items corresponding to each group, following predefined criteria as described (Fernández-Ballart et al., 2010).

Supplementary Table 3. Immune response markers in control and PCR+ groups. An ANOVA test was applied for parametric variables, and the Kruskal-Wallis test was implemented to assess differences between nonparametric ones. p-values were adjusted using the BH correction. Data are expressed as mean ± SD. No statistical differences were found between groups (p-adj < 0.05).

SARS-CoV-2 target	Immune response marker	Control	PCR+ group	p-value	p-adj
Neutralizing Ab anti-S	NT_{50}	124.79 ± 98.5	280.8 ± 516.38	0.80	0.98
Anti-S	% CD107a+/CD8+	0.45 ± 0.64	0.68 ± 1.10	0.98	0.98
	% IFNγ+/CD8+	0.15 ± 0.18	0.27 ± 0.22	0.15	0.90
	% IL2+/CD8+	0.19 ± 0.15	0.18 ± 0.23	0.56	0.98
	% TNFα+/CD8+	0.16 ± 0.15	0.25 ± 0.28	0.77	0.98
Anti-M	% CD107a+/CD8+	0.07 ± 0.06	0.10 ± 0.09	0.49	0.75
	% IFNγ+/CD8+	0.04 ± 0.05	0.02 ± 0.04	0.30	0.90
	% IL2+/CD8+	0.07 ± 0.18	0.03 ± 0.06	0.92	0.98
	% TNFα+/CD8+	0.09 ± 0.13	0.07 ± 0.10	0.86	0.98
Anti-N	% CD107a+/CD8+	0.07 ± 0.06	0.09 ± 0.08	0.73	0.98
	% IFNγ+/CD8+	0.02 ± 0.05	0.02 ± 0.03	0.89	0.98
	% IL2+/CD8+	0.02 ± 0.03	0.01 ± 0.02	0.04	0.75
	% TNFα+/CD8+	0.04 ± 0.08	0.07 ± 0.11	0.30	0.90
Anti-S	% CD69+/CD4+	2.62 ± 2.94	3.32 ± 3.1	0.26	0.90
	% CD134+CD137+/CD4+	0.82 ± 0.48	0.69 ± 0.43	0.49	0.75
	% CD69+CD134+CD137+/CD4+	0.39 ± 0.19	0.37 ± 0.22	0.80	0.80
Anti-RBD	% CD69+/CD4+	2.58 ± 2.26	3.74 ± 2.43	0.21	0.75
	% CD134+ CD137+/CD4+	0.75 ± 0.41	0.61 ± 0.42	0.37	0.75
	% CD69+ CD134+ CD137+/CD4+	0.35 ± 0.16	0.29 ± 0.19	0.67	0.98
Anti-Mpro	% CD69+/CD4+	4.51 ± 2.85	5.37 ± 5.76	0.88	0.98
	% CD134+CD137+/CD4+	0.18 ± 0.21	0.20 ± 0.22	0.94	0.98
	% CD69+ CD134+ CD137+/CD4+	0.11 ± 0.14	0.14 ± 0.14	0.64	0.98
Anti-N	% CD69+/CD4+	3.52 ± 2.46	5.77 ± 5.03	0.30	0.90
	% CD134+ CD137+/CD4+	0.53 ± 0.29	0.58 ± 0.41	0.73	0.80
	% CD69+ CD134+ CD137+/CD4+	0.28 ± 0.18	0.34 ± 0.24	0.54	0.75

Supplementary Table 4. Significant differential pathways obtained by DESeq2 method. To simplify interpretation, a strict cutoff (log2FoldChange > 3.5 or < -3.5) was applied due to the high number of significant pathways identified (4728 pathways with p-adj < 0.05).

Pathway	Higher in	log2FC	padj
Superpathway of branched chain amino acid biosynthesis	Control	-4.45	0.000
UDP-N-acetylmuramoyl-pentapeptide biosynthesis I	Control	-4.43	0.000
Peptidoglycan biosynthesis I	Control	-4.42	0.000
L-isoleucine biosynthesis I	Control	-4.40	0.000
Flavin biosynthesis I	Control	-4.01	0.001
Guanosine ribonucleotides de novo biosynthesis	Control	-3.79	0.000
Folate transformations II	Control	-3.75	0.001
Superpathway of L-lysine, L-threonine, and L-methionine biosynthesis II	Control	-3.70	0.001
Pentose phosphate pathway I	Control	-3.63	0.010
GDP-mannose biosynthesis	PCR+ group	3.51	0.006
Gondoate biosynthesis	PCR+ group	3.53	0.003
L-histidine degradation III	PCR+ group	3.62	0.007
Guanosine ribonucleotides de novo biosynthesis	PCR+ group	4.68	0.000
Inosine-5-phosphate biosynthesis I	PCR+ group	4.85	0.000
Inosine-5-phosphate biosynthesis II	PCR+ group	4.87	0.000
dTDP-β-L-rhamnose biosynthesis	PCR+ group	4.90	0.000
L-arginine biosynthesis II	PCR+ group	5.03	0.000
UMP biosynthesis I	PCR+ group	5.04	0.000
ADP-L-glycero-β-D-manno-heptose biosynthesis	PCR+ group	5.07	0.000
PreQ0 biosynthesis	PCR+ group	5.10	0.000
Myo-, chiro-, and scyllo-inositol degradation	PCR+ group	5.18	0.000
UMP biosynthesis II	PCR+ group	5.19	0.000
UMP biosynthesis III	PCR+ group	5.19	0.000
Coenzyme A biosynthesis I	PCR+ group	5.21	0.000
L-methionine biosynthesis III	PCR+ group	5.23	0.000
Folate transformations II	PCR+ group	5.25	0.000
L-isoleucine biosynthesis III	PCR+ group	5.25	0.000
Chorismate biosynthesis I	PCR+ group	5.25	0.000
Chorismate biosynthesis from 3-dehydroquinate	PCR+ group	5.25	0.000
L-lysine biosynthesis III	PCR+ group	5.33	0.000
L-methionine biosynthesis IV	PCR+ group	5.38	0.000
Tetrapyrrole biosynthesis I	PCR+ group	5.40	0.000
Queuosine biosynthesis I	PCR+ group	5.52	0.000
S-adenosyl-L-methionine salvage I	PCR+ group	6.05	0.000
Chorismate biosynthesis I	PCR+ group	6.24	0.000
Coenzyme A biosynthesis I	PCR+ group	6.34	0.000
Chorismate biosynthesis from 3-dehydroquinate	PCR+ group	6.36	0.000
L-lysine biosynthesis VI	PCR+ group	6.52	0.000
5-aminoimidazole ribonucleotide biosynthesis I	PCR+ group	6.91	0.000
5-aminoimidazole ribonucleotide biosynthesis II	PCR+ group	6.91	0.000
Superpathway of 5-aminoimidazole ribonucleotide biosynthesis	PCR+ group	6.91	0.000
Glycogen biosynthesis I	PCR+ group	6.99	0.000
L-ornithine biosynthesis I	PCR+ group	7.08	0.000
L-arginine biosynthesis I	PCR+ group	7.14	0.000
L-arginine biosynthesis II	PCR+ group	7.16	0.000

Supplementary Table 5. Concentration (mM) of short-chain fatty acids (SCFAs) in control and PCR+ groups. Parametric variables (acetic, butyric, valeric, caproic, and total SCFAs) were evaluated through an ANOVA test. Nonparametric features (propionic, isobutyric, and isovaleric) were assessed by the Kruskal-Wallis test. All p-values were adjusted by BH (FDR < 0.05). No statistical differences were found between groups.

SCFAs	Control	PCR+ group	p-value	p-adj
Acetic	46.71 ± 19.63	46.85 ± 26.86	0.99	0.99
Propionic	16.08 ± 8.04	13.37 ± 11.33	0.28	0.28
Isobutyric	1.57 ± 0.67	1.41 ± 0.83	0.24	0.28
Butyric	14.36 ± 7.64	10.45 ± 8.33	0.21	0.52
Isovaleric	2.75 ± 1.20	2.36 ± 1.53	0.17	0.28
Valeric	2.18 ± 0.83	1.54 ± 0.97	0.07	0.37
Caproic	2.35 ± 1.17	2.60 ± 0.82	0.79	0.99
Total SCFAs	85.33 ± 35.04	76.33 ± 45.56	0.57	0.95
